# Irreversible depletion of intestinal CD4^+^ T cells is associated with T cell activation during chronic HIV infection

**DOI:** 10.1172/jci.insight.146162

**Published:** 2021-11-22

**Authors:** Osaretin E. Asowata, Alveera Singh, Abigail Ngoepe, Nicholas Herbert, Rabiah Fardoos, Kavidha Reddy, Yenzekile Zungu, Faith Nene, Ntombifuthi Mthabela, Dirhona Ramjit, Farina Karim, Katya Govender, Thumbi Ndung’u, J. Zachary Porterfield, John H. Adamson, Fusi G. Madela, Vukani T. Manzini, Frank Anderson, Alasdair Leslie, Henrik N. Kløverpris

**Affiliations:** 1 Africa Health Research Institute (AHRI), Durban, South Africa.; 2 School of Laboratory Medicine and Medical Sciences, University of KwaZulu-Natal, Durban, South Africa.; 3 Department of Immunology and Microbiology, University of Copenhagen, Denmark.; 4 University College London, Division of Infection and Immunity, London, United Kingdom.; 5 Max Planck Institute for Infection Biology, Berlin, Germany.; 6 Department of Microbiology, Immunology, and Molecular Genetics, University of Kentucky, Lexington, Kentucky, USA.; 7 Division Upper Gastrointestinal Tract and Colorectal Surgery, Inkosi Albert Luthuli Central Hospital (IALCH), University of KwaZulu-Natal, Durban, South Africa.

**Keywords:** AIDS/HIV, Gastroenterology, T cells

## Abstract

HIV infection in the human gastrointestinal (GI) tract is thought to be central to HIV progression, but knowledge of this interaction is primarily limited to cohorts within Westernized countries. Here, we present a large cohort recruited from high HIV endemic areas in South Africa and found that people living with HIV (PLWH) presented at a younger age for investigation in the GI clinic. We identified severe CD4^+^ T cell depletion in the GI tract, which was greater in the small intestine than in the large intestine and not correlated with years on antiretroviral treatment (ART) or plasma viremia. HIV-p24 staining showed persistent viral expression, particularly in the colon, despite full suppression of plasma viremia. Quantification of mucosal antiretroviral (ARV) drugs revealed no differences in drug penetration between the duodenum and colon. Plasma markers of gut barrier breakdown and immune activation were elevated irrespective of HIV, but peripheral T cell activation was inversely correlated with loss of gut CD4^+^ T cells in PLWH alone. T cell activation is a strong predictor of HIV progression and independent of plasma viral load, implying that the irreversible loss of GI CD4^+^ T cells is a key event in the HIV pathogenesis of PLWH in South Africa, yet the underlying mechanisms remain unknown.

## Introduction

HIV-1–associated alterations within the gastrointestinal (GI) tract were first described in 1984 by Kotler and colleagues, who reported histologic abnormalities in the GI mucosa of infected patients (1, 2). HIV-1 infection and replication is far more prevalent in the CD4^+^ T cells of the GI mucosa (3–6) than in blood CD4^+^ T cells (5, 7–9), leading to rapid and severe loss of GI CD4^+^ T cells following infection. The resulting intestinal damage and microbial translocation (10) is thought to precipitate systemic inflammation and chronic immune activation, which ultimately drives disease progression (10). Crucially, these events are not fully reconstituted by antiretroviral treatment (ART) (11–15) and may have long-term health consequences for the growing number of people living with HIV (PLWH) on ART. This includes an elevated risk of noninfectious comorbidities (16, 17), such as cardiovascular disease, renal failure, liver disease, neurocognitive disease, osteoporosis, diabetes, and gut-associated cancers (17–23). However, the direct link between HIV-associated intestinal epithelial barrier break down, CD4 loss in the GI tract, and HIV pathology has yet to be established, representing a major hindrance in the development of treatment strategies to restore gut homeostasis.

The intestine is the largest outward-facing organ in the body, and it contains more activated CD4^+^ T cells and tissue-resident HIV reservoirs than any other human tissue site (24–26). Persistent viral replication within the gut HIV reservoir and high levels of HIV DNA in the gut CD4^+^ memory T cells are major obstacles for HIV eradication (6, 27). ART initiation during early acute HIV-1 infection reduces immune activation and limits reservoir size, but it does not appear to prevent its formation or lead to its eradication after prolonged treatment (26, 28–30). Reasons for these issues are not clear, but they may include suboptimal antiretroviral (ARV) drug penetration into the gut mucosa (14, 27, 31, 32). Indeed, recent data suggest that some regions of the gut may have little or no exposure to antiretroviral drugs during ART (33).

The majority of studies investigating HIV-related gut immunopathology have been conducted on White populations and/or carried out in Westernized countries. Intestinal health and immunity, however, is influenced by genetics, diet, microbiome, and comorbidities; thus, it varies greatly between different populations. Therefore, the immunopathology of HIV in the GI tract of these study populations may be distinct from Sub-Saharan African populations, where two-thirds of the global HIV population live. In addition, for practical reasons, previous work has focused primarily on the large intestine. As a result, the impact of HIV infection on the small intestine, where the immune composition, physiology, and microbiome are distinct, remains less understood (34–37).

Here, we present data collected from a large cohort of more than 500 study subjects undergoing clinically indicated endoscopy or colonoscopy who were recruited from extremely high HIV-1 endemic areas within KwaZulu-Natal (KZN), South Africa. The HIV-infected participants presented to the GI clinic at a younger age, and gut pinch biopsies obtained from their colon, rectum, and duodenum revealed severe and irreversible HIV-associated CD4^+^ T cell depletion — in particular, within the small intestine. This was associated with persistent HIV expression, as shown by histological staining of HIV-p24 in gut CD4^+^ T cells. This was greatest in the colon, in which CD4 depletion was less severe, suggesting that target cell availability may affect reservoir size in the GI tract, in addition to the poor penetration of ART drugs observed. The degree of GI CD4^+^ T cell depletion is directly correlated with immune activation of blood CD4^+^ T cells only in PLWH, which strongly predicts HIV disease progression, independent of viral load (38). Plasma markers of systemic immune activation, however, were elevated in all study participants and did not correlate with GI CD4^+^ T cell levels, suggesting that these biomarkers are not directly linked to HIV pathology per se. This cohort represents a large and unique resource to further study intestinal health and HIV reservoir dynamics in PLWH from areas of rural Sub-Saharan Africa, which sits at the center of the global HIV epidemic.

## Results

### Diverse GI diagnoses and increased risk of biliary pathology in PLWH.

To study the impact of HIV on the intestinal mucosa, we recruited 529 adult participants from KZN, South Africa, presenting in the GI clinic for either endoscopy, including ERCP (small intestine) or colonoscopy (large intestine) procedures between 2015 and 2019. Participants were from area of high HIV endemicity, and 170 (32%) were infected with HIV (Table 1). In total, 84% of HIV infected individuals had undetectable plasma HIV viral load due to ART, with HIV diagnosed for a median of 6.3 years — and were reportedly on ART for 5 years, with median CD4 counts of 647 cells/mm^3^. The 29 HIV-infected individuals with detectable viremia (viral load [VL] > 20 RNA copies/mL plasma) were diagnosed for a median of 0.3 years with CD4^+^ T cell counts of 297 cells/mm^3^ and median viral load of 29,350 RNA copies/mL plasma (Table 1). Less than 3% presented with active Tuberculosis (TB) infection, but previous TB infections were enriched in HIV-infected individuals (4% versus 24%, *P* < 0.0001) (Supplemental Table 1; supplemental material available online with this article; https://doi.org/10.1172/jci.insight.146162DS1), consistent with increased risk of TB infection in HIV-infected individuals.

**Figure 1:**
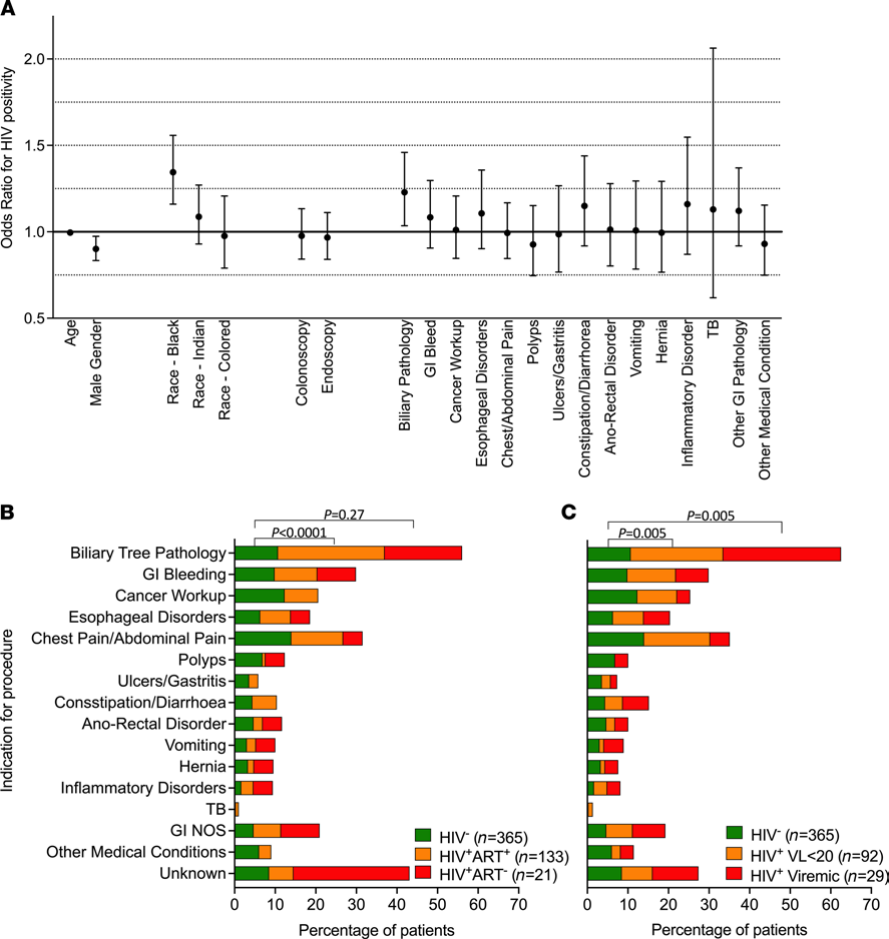
Surgical indications of participants in GI study cohort. (**A**) Odds ratios for HIV positivity associated with demographic variables and procedural indications. (**B**) Surgical indications stratified by HIV and ART status (*n* = 519). (**C**) Surgical indications stratified by HIV and viral load status (*n* = 486). Note: colored is a term used in South Africa, including on the national census, for persons of mixed-race ancestry who developed a distinct cultural identity over several hundred years.

In this cohort, clinical diagnoses were dominated by noninfectious morbidities such as biliary pathology, GI bleed, esophageal disorders, and pain within the chest and abdomen (Supplemental Figure 1). The most common indications for colonoscopy were cancer, polyps, constipation/diarrhea, and ano-rectal disorders, while for upper endoscopies, these were dominated by biliary, esophageal, and abdominal complications. In 45 participants (9%), no obvious GI-related diagnoses following investigation were identified (Supplemental Figure 1). Stratifying participants based on HIV status showed that HIV positivity was enriched or highest among female and black participants in the HIV positive group, consistent with the demographic of the HIV epidemic in this region (Figure 1A). Overall, the GI complication of biliary pathology was enriched or highest in the HIV^+^ group (OR 1.2, 25.3% [39 of 154]; *P* = 0.02) by multivariate analysis (Figure 1A). Stratifying clinical diagnoses by ART status revealed that biliary pathology was enriched in ART-treated (OR 3.0; 95% CI, 1.8–5.0; *P* < 0.0001) but not in ART-untreated individuals (OR 2.0; 95% CI, 0.7–5.7; *P* = 0.27) (Figure 1B), while both HIV plasma virally suppressed and viremic participants showed consistent enrichment for biliary pathology (both OR 2.5; 95% CI, 1.4–4.3; *P* = 0.0050) (Figure 1C). Taken together, these clinical diagnoses show a diverse spectra of GI complications, of which frequencies of inflammatory disorders — including inflammatory bowel disease, diverticulitis, prostatitis, ulcerative colitis, and inflammation not otherwise specified — were overall less frequent to those observed in White cohorts (39) and highlight distinct GI-related complications in this Sub-Saharan African cohort. However, with the exception of biliary pathology, clinical presentation of HIV-infected and -uninfected subjects was similar and, therefore, unlikely to significantly skew the comparison of these groups.

### Early presentation of GI-related complications in HIV-infected individuals.

**Figure 2:**
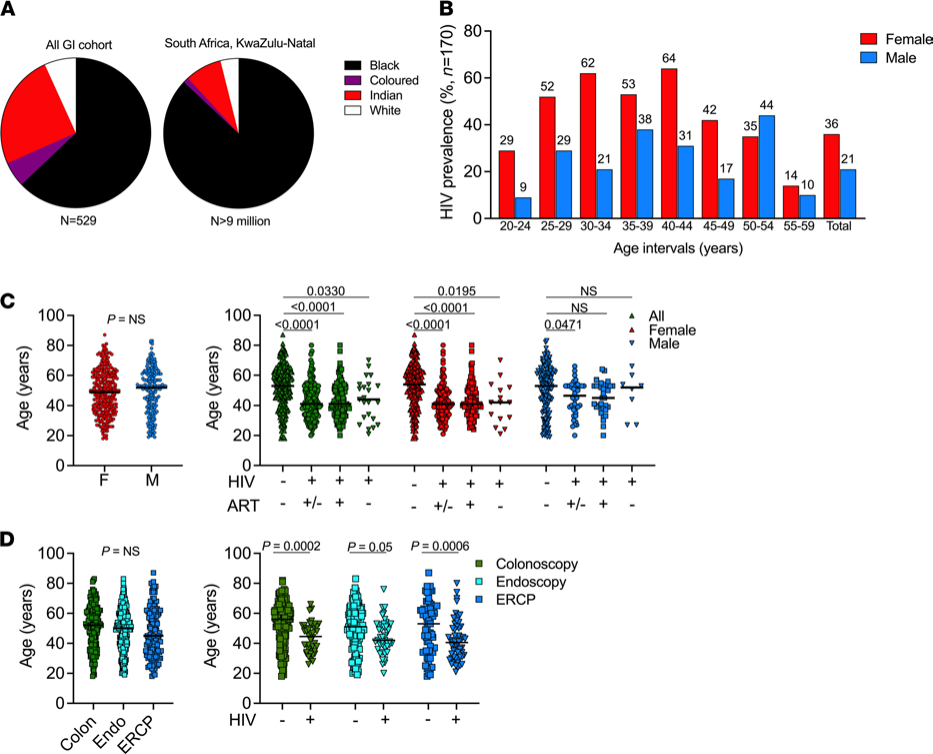
Characteristics of study participants. (**A**) Study participants (*n* = 529) stratified by race and ethnicity compared with the general population of the KwaZulu-Natal province. Note: colored is a term used in South Africa, including on the national census, for persons of mixed-race ancestry who developed a distinct cultural identity over several hundred years. (**B**) HIV prevalence stratified by age group and sex, female (red) and male (blue) (*n* = 170). (**C**) Age of presentation to the GI clinic stratified by gender, female (red) and male (blue); HIV prevalence and ART status stratified by age and sex. All participants, green; female, red; male, blue. *P* values by Mann-Whitney *U* test and Kruskal-Wallis test. (**D**) Participants age of presentation for GI surgical procedures irrespective of HIV-1 status. Participants’ age of presentation for GI surgical procedure stratified by HIV-1 status. *P* values by Kruskal-Wallis test.

The HIV epidemic in South Africa is known to be discordant for ethnicity, age, and sex (41–43). We therefore stratified our cohort by race and ethnicity and found a skewed distribution of those not of Black race compared with the background provincial data, which is likely to be at least in part explained by differential access to tertiary healthcare units, in addition to potential differences in genetic and environmental factors (Figure 2A and Supplemental Table 1). Overall, HIV prevalence was higher in women, reaching > 60% infection in the 25–44 year age group (Figure 2B); this is consistent with recent population level studies from the same region (40, 41). Strikingly, while females and males presented to the clinic for GI complications at a similar age, HIV-infected participants presented a median of 12 years earlier than HIV-1–uninfected participants (41 years [IQR 34–52 years] versus 53 years [IQR 40–63 years]; *P* < 0.0001) (Figure 2C), irrespective of ART treatment status, with females and males presenting 13 and 6.5 years younger, respectively. We found no age differences within the 3 indicated procedures (colonoscopy, upper endoscopy, and endoscopic retrograde cholangiopancreatograpgy (ERCP), but in each procedure, HIV infection was associated with an overall younger population (Figure 2D).

In a subset of HIV-infected participants, full blood parameters were available, including WBC and RBC analysis. Compared with HIV-uninfected controls, this revealed reduced CD4^+^ T cell (median of HIV uninfected [858] versus HIV infected virally suppressed [641] versus HIV infected viremic [297] cells/μL) and elevated CD8 T cells (median of CD8 T cells from HIV uninfected [610] versus HIV infected virally suppressed [670] versus HIV infected viremic [1040] cells/μL) counts in both virally suppressed and viremic groups compared with HIV uninfected participants (Supplemental Figure 2). In addition, HIV infection was associated with elevated neutrophils and basophils, and with increased neutrophil/lymphocyte ratio (NLR), suggesting overall increased inflammation irrespective of viral suppression (Supplemental Figure 2A). Analysis of platelets and RBC parameters showed significant changes, including reduced RBC counts with skewed width distribution and volume, and reduced mass and hemoglobulin levels (Supplemental Figure 2B). These data are consistent with HIV-1 infection leading to GI pathology in Sub-Saharan African cohorts (40, 41) and suggest that GI complications may arrive earlier in HIV-infected individuals with reduced CD4^+^ T cell counts and overall increased systemic inflammation.

### Irreversible depletion of duodenal CD4^+^ T cells despite long-term ART.

**Figure 3:**
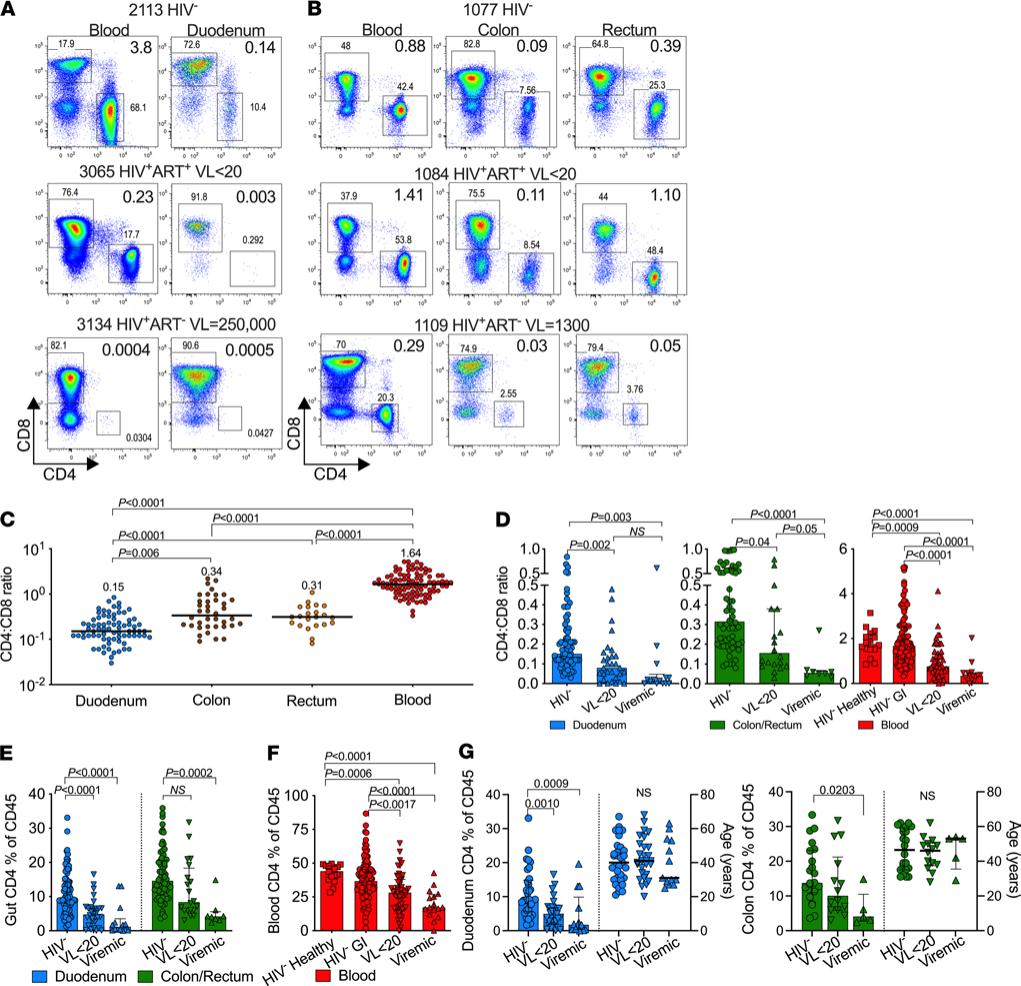
Irreversible depletion of duodenal mucosal CD4^+^ T cells despite long-term ART. (**A**) Gating strategy to identify CD4^+^ and CD8^+^ T cells in patient-matched blood and duodenum from HIV-uninfected and -infected participants. (**B**) Gating strategy to identify CD4^+^ and CD8^+^ T cells in participants-matched blood, colon, and rectum from HIV-uninfected and -infected patients. (**C**) CD4/CD8 ratios of duodenum, colon, rectum, and blood from HIV-uninfected participants. *P* values by Kruskal-Wallis test. (**D**) CD4/CD8 ratios of duodenum (blue), colon/rectum (green), and blood (red) from HIV-uninfected, HIV-1–infected suppressed, and viremic participants. *P* values by Kruskal-Wallis test. (**E**) Percentage of CD4^+^ T cell frequencies of CD45^+^ cells from the duodenum (blue) and colon/rectum (green) of HIV-1–uninfected, -infected virally suppressed, and viremic participants. (**F**) Percentage of CD4^+^ T cell frequencies of CD45^+^ cells from blood (red) of healthy HIV-uninfected non-GI study participants, HIV-uninfected GI study participants, HIV-1–infected suppressed, and viremic participants from our GI cohort. *P* values by Kruskal-Wallis test. (**G**) Duodenum (blue) and colon (green) percentage of CD4^+^ T cell frequencies of CD45^+^ cells stratified by age from HIV-uninfected GI study participants, HIV-1–infected suppressed, and viremic participants from our GI cohort. *P* values by Kruskal-Wallis test.

**Figure 4:**
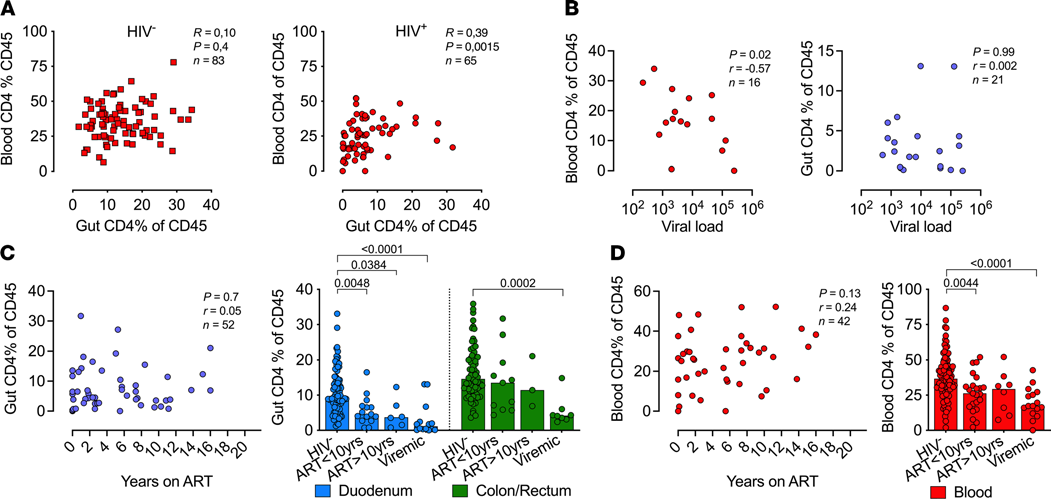
Depleted duodenal CD4^+^ T cells fails to reconstitute even after 10 years of ART. (**A**) Correlation of percentage of CD4^+^ T cell frequencies of CD45^+^ cells of pooled gut tissues with that of blood among HIV-uninfected and -infected participants. (**B**) Correlation of viral load with CD4% of CD45 cells of blood (red) and gut tissues (blue). (**C**) Correlation of participants’ duration on ART (years) with percentage of CD4^+^ T cell frequencies of CD45^+^ cells of gut tissues (blue) and of percentage of CD4^+^ T cell frequencies of CD45^+^ cells of duodenum (blue) and colon/rectum (green) tissues stratified by HIV and ART status. (**D**) Correlation of participants’ duration on ART (years) with percentage of CD4^+^ T cell frequencies of CD45^+^ cells of blood (red), correlations by spearman test and with percentage of CD4^+^ T cell frequencies of CD45^+^ cells of blood stratified by HIV and ART status. Correlations by spearman test and *P* values by Kruskal Wallis test for multiple comparisons.

Next, to investigate alterations in CD4^+^ and CD8^+^ T cell homeostasis within the small and large intestine during HIV-1 infection, we quantified T cells from fresh duodenum, colon, and rectum biopsies in 228 study participants (Figure 3, A and B). The CD4/CD8 T cell ratios in the GI tract of HIV-uninfected individuals were overall lower than those found in matched uninfected blood samples, and they showed compartment-specific differences between the small (duodenum) and large (colon/rectum) intestine (*P* < 0.01) but similar ratios between colon and rectum (Figure 3C) (42). HIV-1 infection reduced CD4/CD8 T cell ratios within the duodenum, colon, and blood that was not restored by full ART-mediated viral suppression in plasma (Figure 3D). Consistent with altered CD4/CD8 T cell ratios, the frequency of CD4^+^ T cells, as a percentage of CD45^+^ cells, was significantly reduced in the duodenum, the colon/rectum, and blood of viremic participants compared with HIV-uninfected participants (*P* < 0.001) (Figure 3E). The CD4 depletion remained highly significant in the duodenum of virally suppressed participants (*P* < 0.001), but it was less pronounced in the colon and no longer reached statistical significance (*P* = 0.1). The blood compartment overall showed consistent CD4 depletion (see Supplemental Figure 2A), although the median change was lower than that observed in the colon for virally suppressed individuals (Figure 3F). Duodenal CD4^+^ T cell depletion, irrespective of plasma viral suppression, remained significant when we controlled for age, sex, and race and ethnicity with overall less impact in the large intestine (Figure 3G). Direct comparison of CD4^+^ T cell levels in blood and duodenum/colon combined showed significant positive correlation in HIV infected (ART^+/–^) participants (*r* = 0.39; *P* = 0.0015) but not among HIV^–^ participants (*r* = 0.10; *P* = 0.4) (Figure 4A). In HIV viremic individuals, we found a strong negative correlation between plasma VL and blood CD4 levels (*r* = **–**0.57; *P* = 0.02) but not between plasma viral loads and gut CD4 levels (*r* = 0.002; *P* = 0.99) (Figure 4B). To explore the disconnect between plasma viremia and GI CD4^+^ T cells further, we used available treatment initiation data from the GI clinic and found no overall correlation between gut CD4^+^ T cell levels and ART duration (*r* = 0.05; *P* = 0.7) (Figure 4C). Breaking this down by compartment, we observed a failure to reconstitute CD4^+^ T cells in the duodenum even after 10 years of ART (2.5-fold reduction versus HIV-uninfected; *P* = 0.04) (Figure 4C). The same trend was not observed in the colon, which might indicate improved reconstitution in this compartment, although fewer samples were available for this analysis. Blood CD4^+^ T cell levels showed a trend toward CD4 restoration (*r* = 0.24; *P* = 0.13) with only a 1.3-fold reduction (*P* = 0.2) in participants receiving ART for more than 10 years (Figure 4D), and this is consistent with a 1.1 reduction in absolute CD4^+^ T cell counts (HIV^–^ versus HIV^+^ VL < 20 ART > 10 years, median 858 versus 795 cells/mm^3^, *P* = 0.99). Taken together, CD4^+^ T cell frequencies within the small and large intestine from > 200 GI patients show that long-term ART fails to restore CD4^+^ T cell levels in the small intestine despite long-term and fully suppressive ART.

### Compartment-specific HIV-1-p24 detection and CD4 depletion in the gut mucosa of ART-treated individuals.

**Figure 5:**
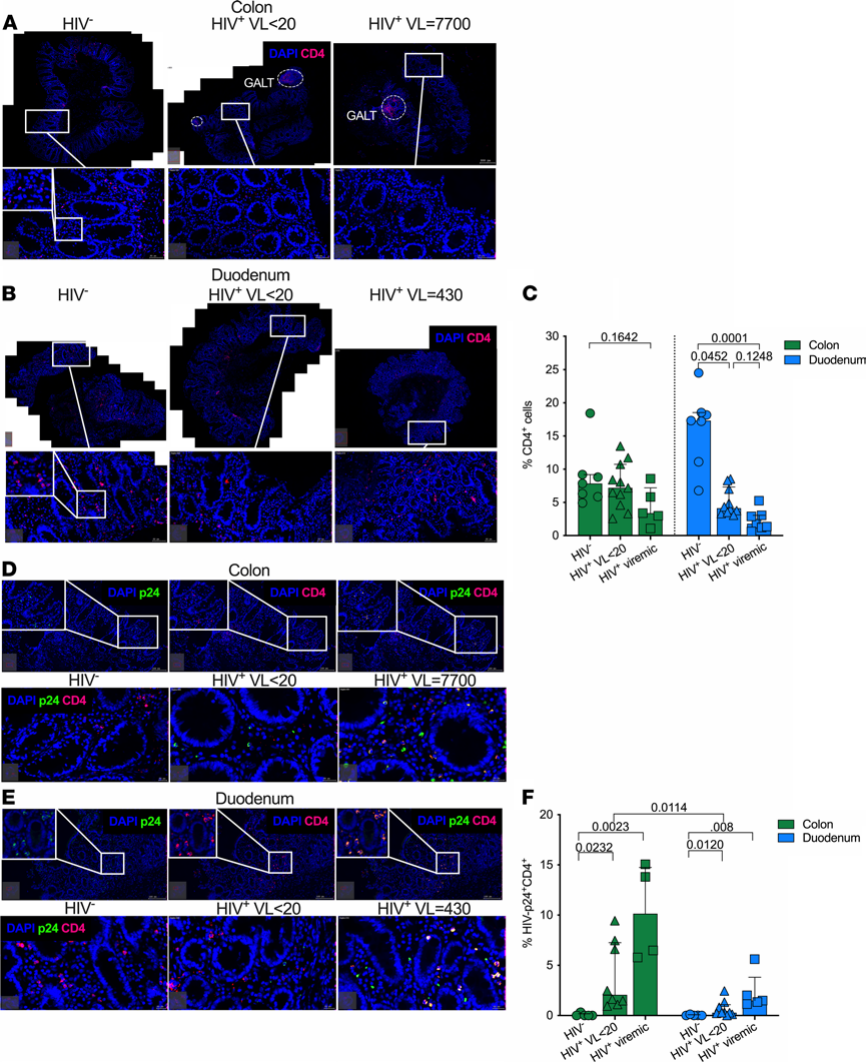
Compartment-specific HIV-1–p24 detection and CD4 depletion in the gut mucosa of ART-treated individuals. (**A**) Representative fluorescent IHC image of CD4^+^ T cells (red) and proportion of total cells stained with DAPI (blue) in colon tissue sections from HIV-uninfected, HIV-infected plasma virally suppressed, and viremic participants. Scale bars: 100 μm for full image and 50 μm and 20 μm for large and small inserted images, respectively. (**B**) Representative fluorescent IHC image of CD4^+^ T cells (red) and proportion of total cells stained with DAPI (blue) in duodenum tissue sections from HIV-uninfected, HIV-infected plasma virally suppressed, and viremic participants. Scale bars: 100 μm for full image and 50 μm and 20 μm for large and small inserted images, respectively (**C**) Quantification of CD4^+^ T cells in colon (green) and duodenum (blue). Each dot is representative of a gut tissue section per participants. *P* values by Kruskal Wallis test corrected for multiple comparisons. (**D**) Representative fluorescent IHC image of colon tissues showing HIV-p24 (green), CD4^+^ T cells (red), and DAPI (blue) from HIV-uninfected, virally suppressed, and viremic participants. Scale bars: 100 μm for full image and 50 μm and 20 μm for large and small inserted images, respectively. (**E**) Representative fluorescent IHC image of duodenum tissues showing HIV-p24 (green), CD4^+^ T cells (red), and DAPI (blue) from HIV-uninfected, virally suppressed, and viremic participants. Scale bars: 100 μm for full image and 50 μm and 20 μm for large and small inserted images, respectively. (**F**) Quantification of HIV-p24 and CD4^+^ T cell proportion of total cells in colon (green) and duodenum (blue) stained with DAPI from HIV-uninfected, virally suppressed, and viremic participants. *P* values by Mann-Whitney *U* test.

Studies of non–Sub-Saharan African cohorts show that CD4^+^ T cells within the large intestinal mucosa are major sites for viral infection and replication even during plasma viral suppression (4, 43). We first quantified CD4-expressing cells in the colon, after exclusion of gut associated lymphoid tissues-like structures (Figure 5A), and in the duodenum tissue sections (Figure 5B). We found severe depletion of duodenal CD4 cells in both virally suppressed (*P* < 0.05) and viremic (*P* = 0.0001) participants, corresponding to 4- and 10-fold reduction, respectively, compared with HIV-uninfected GI participant controls (Figure 5C); however, we found no significant CD4 depletion in the colon. This is consistent with our findings from flow cytometric analysis of these compartments with severe CD4^+^ T cell depletion — in particular, that observed within the duodenum (Figure 3).

Next, we tested HIV-1 persistence in the gut by costaining tissue sections for HIV-p24 and CD4 (Figure 5, D and E, and Supplemental Figure 3A). We found HIV-p24 protein in both the duodenum and colon tissues of both virally suppressed and viremic individuals that colocalized with CD4 expression and most often within the lamina propria (Figure 5, D and E). We detected more HIV-p24 protein in the colon of both plasma virally suppressed and viremic individuals compared with that of the duodenum compartment, suggesting that compartment-specific differences exist in the gut for HIV detection (Figure 5F). We confirmed HIV detection by digital droplet PCR (ddPCR) and found a trend toward more HIV DNA in the duodenum and colon compared with blood, despite low overall CD4 frequencies in the gut compared with the blood compartment (Supplemental Figure 3B). To examine the relative contribution of CD4-expressing macrophages to the overall HIV-p24–expressing cells, we included CD68 staining and found relatively high frequencies of CD4 and CD68–coexpressing cells and with more HIV-p24^+^CD68^+^ cells coexpressing CD4 in the colon compartment compared with the duodenum, suggesting that macrophages also may contribute to overall increased level of HIV-p24 in the colon, at least in the plasma virally suppressed participants (Supplemental Figure 3, C–E). Thus, we found higher HIV-p24 expression in the colon and lower target cell availability in the duodenum in both suppressed and viremic participants, suggesting that compartment-specific effects may contribute to the dynamics of the HIV reservoir in the gut.

### Poor ARV mucosal drug penetration levels in the GI tract does not explain compartment-specific differences in PLWH.

**Figure 6:**
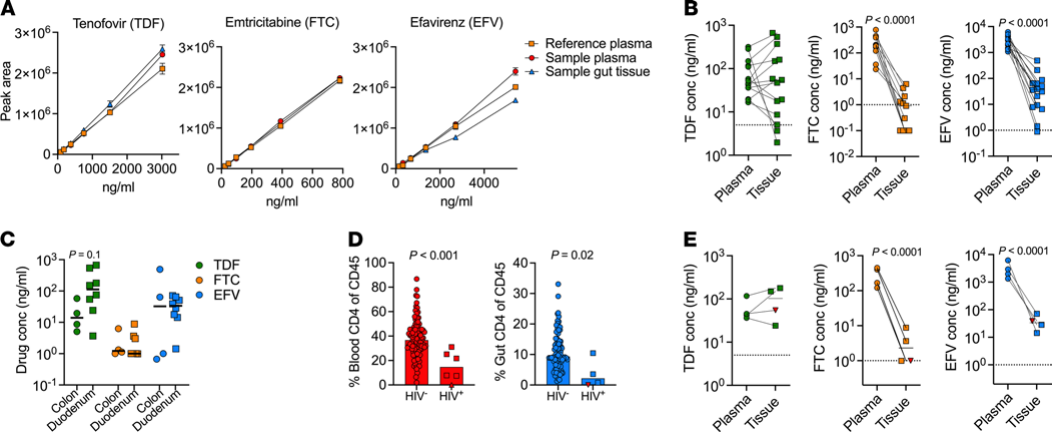
ARV drug concentration within plasma and intestinal mucosal tissue sites. (**A**) Quantitation of ARV drugs (TDF, FTC, and EFV) in gut tissue and plasma using LC-MS/MS, showing biological matrix equivalency between spiked plasma and gut tissue samples. (**B**) Comparison of TDF, FTC, and EFV concentrations between gut tissue (normalized by wet tissue mass) and patient-matched plasma with dotted line showing limit of detection. (**C**) ARV quantification stratified by colon and duodenum tissue. (**D**) CD4% of CD45 lymphocytes in blood (red) and tissue (blue) of participants whose ARV concentration was tested, stratified by HIV status. (**E**) TDF, FTC, and EFV concentrations between gut tissue (normalized by wet tissue mass) and patient-matched plasma in subset of participants with available percentage of CD4^+^ T cell frequencies of CD45^+^ cells, with dotted line showing limit of detection. TDF, Tenofovir; FTC, Emtricitabine; EFV, Efavirenz.

Next, we tested if the mucosal HIV-p24 protein detection and CD4^+^ T cell depletion in the presence of plasma viral suppression could be linked to poor ARV penetration into the GI tissue. For this, we established a liquid chromatography–tandem mass spectrometry (LC-MS/MS) approach to accurately quantify the concentration of the first-line ART regimen drugs used at the time of study (44); Tenofovir (TDF), Emtricitabine (FTC), Efavirenz (EFV), in fresh mucosal tissue and matched plasma (Figure 6A). Using this methodology, we observed significantly lower concentrations of FTC (*P* < 0.0001) and EFV (*P* < 0.0001), but not TDF, in intestinal tissue compared with paired plasma, when normalizing by tissue mass (see Methods) (Figure 6B). In participants with detectable plasma ARV, FTC was only detected in 45% (5 of 11) tissue samples, compared with 86% (12 of 14) and 93% (14 of 15) for TDF and EFV, respectively (Figure 6B). Importantly, however, levels of all 3 drugs were not different between the duodenum and colon compartments (Figure 6C). Moreover, in a subset of participants for whom the percentages of CD4^+^ T cell frequencies and drug levels were available, we consistently found CD4^+^ T cell depletion in both blood and tissue (Figure 6D), despite detection of all 3 ARVs in both compartments (Figure 6E).

Although, it is not clear from these data whether the reduced levels of FTC and EFV directly contribute to persistent viral replication or CD4 depletion in the GI tract, they do indicate that the differences in CD4^+^ T cell depletion and HIV-p24 detection between the duodenum and colon are not consequences of differential ARV drug penetration into these 2 distinct gut compartments. These data suggest that penetration of some ARV drugs into the gut mucosa is reduced but that this alone does not explain the lack of CD4^+^ T cell reconstitution observed within the small intestine.

### Persistent systemic T cell activation correlates with gut CD4^+^ T cell depletion only in PLWH.

**Figure 7:**
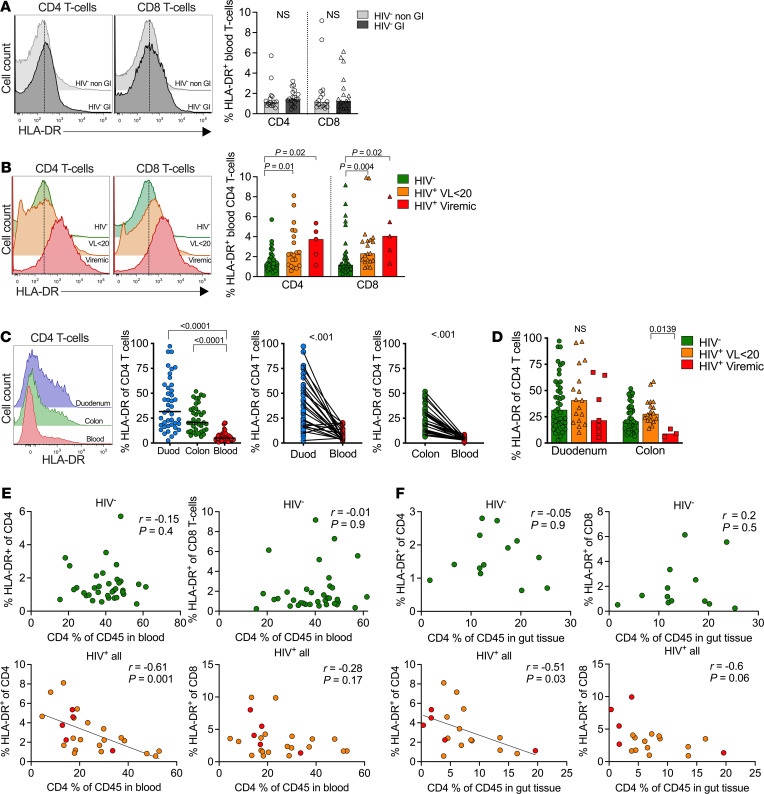
Persistent systemic T cell activation correlates with gut CD4^+^ T cell depletion. (**A**) Gating strategy to identify CD4^+^ and CD8^+^ T cell activation marker, HLA-DR in blood, comparing HIV-uninfected non-GI and GI participants. (**B**) T cell activation (CD4^+^HLA-DR^+^ and CD8^+^HLA-DR^+^) in blood from GI cohort participants stratified by viral load. *P* values by Kruskal-Wallis test. (**C**) CD4^+^ T cell activation in duodenum (blue), colon (green), and blood (red) from HIV-uninfected GI participants. *P* values by Kruskal-Wallis test. (**D**) CD4^+^ T cell activation in HIV-uninfected GI participants (green) compared with HIV-1–infected virally suppressed (orange) and viremic (red) participants from duodenum and colon. *P* values by Kruskal-Wallis test. (**E**) Correlations of percentage of CD4^+^ T cell frequencies of CD45^+^ cells in blood with percentage of CD4^+^ and CD8^+^ T cell activation among HIV-uninfected and HIV-infected participants. (**F**) Correlations of percentage of CD4^+^ T cell frequencies of CD45^+^ cells in gut tissues with percentage of CD4^+^ and CD8^+^ T cell activation among HIV-uninfected and HIV-infected participants. *P* and *r* values by Pearson correlations.

Several studies report that both persistent T cell activation and systemic inflammation, despite ART viral suppression, are linked to increased risk of morbidity and mortality in PLWH (43, 45–49). Therefore, to test the association between GI CD4^+^ T cell depletion and activation, as well as systemic T cell activation in this GI cohort, we measured HLA-DR expression as a surrogate marker for T cell activation by flow cytometry (Figure 7A). As all GI participants are undergoing medical examination (Figure 1), we compared HLA-DR expression in the blood of HIV^–^ participants from the GI cohort with that of healthy non-GI HIV^–^ donors from an unrelated control cohort (42) and found no significant differences (Figure 7A). By contrast, HLA-DR expression was upregulated on blood CD4 and CD8 T cell subsets in both HIV virally suppressed participants and viremic participants, confirming HIV driven T cell activation in these individuals (Figure 7B). Next, we measured HLA-DR expression on GI CD4^+^ T cells (Supplemental Figure 4) and found overall higher steady state expression compared with the blood compartment (*P* < 0.001) (Figure 7C), which is expected, given the persistent exposure of GI T cells to the gut microbiome. Surprisingly, we did not see significantly increased HLA-DR expression on duodenum or colon CD4^+^ T cells in the virally suppressed individuals, and activation was actually lower in the viremic group, reaching significance in the colon (*P* = 0.01) (Figure 7D).

Next, we tested the relationship between blood T cell activation and CD4^+^ T cell frequency, and we found a direct inverse correlation in the blood of HIV-infected individuals with CD4^+^ T cell activation (*r* = –0.61; *P* = 0.001) that was not observed in the HIV uninfected group (*r* = –0.01; *P* = 0.9) or for CD8 T cell activation in either group (Figure 7E). Next, we correlated HLA-DR expression on blood CD4 and CD8 T cells to mucosal CD4^+^ T cell frequency and consistently found a strong inverse correlation in HIV-infected individuals with activation of both CD4^+^ T cells (*r* = –0.51; *P* = 0.03) and CD8 T cells in the blood (*r* = –0.61; *P* = 0.06) (Figure 7F). Again, this trend was not observed in HIV uninfected participants (*r* = –0.05, *P* = 0.9; *r* = 0.2, *P* = 0.5). Therefore, these data suggest a potential mechanistic link between HIV-mediated CD4^+^ T cell depletion in the GI tract and systemic CD4 and CD8 T cell activation, which is a strong predictor of HIV disease progression independent of viral load (51).

### Elevated intestinal and inflammatory markers are not specific to HIV GI pathology and do not correlate with HIV-associated depletion of gut CD4^+^ T cells.

**Figure 8:**
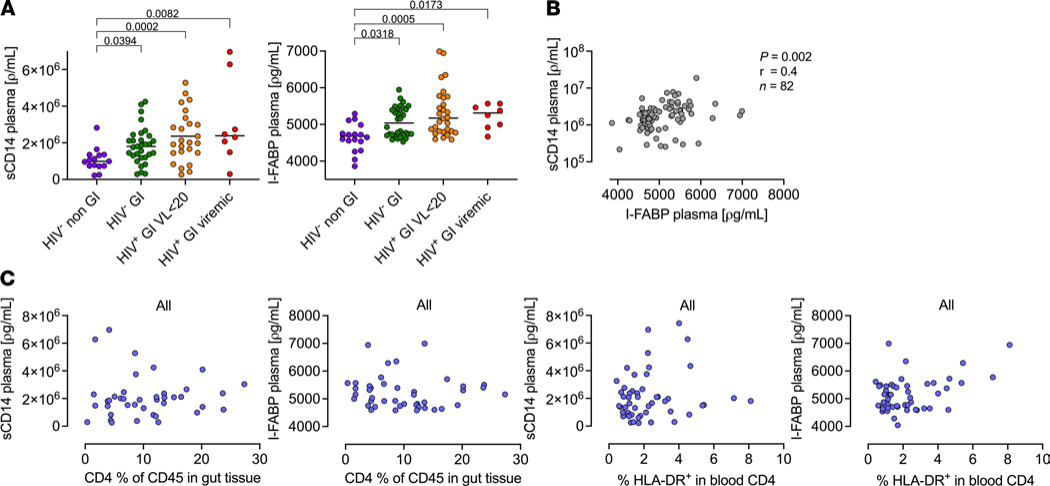
Elevated intestinal and inflammatory markers are associated with GI pathology but do not correlate with HIV-associated depletion of gut CD4^+^ T cells. (**A**) Increased sCD14 and I-FABP among HIV-1–infected virally suppressed (orange) and viremic participants (red) compared with healthy HIV-uninfected participants (purple). *P* values by Kruskal-Wallis test. (**B**) sCD14 correlates positively with I-FABP. *P* and *r* values by Pearson correlations. (**C**) Lack of correlation between percentage of CD4^+^ T cell frequencies of CD45^+^ cells in gut tissues with plasma sCD14 and I-FABP; there is a lack of correlation between percentage of activated CD4 in the blood with plasma sCD14 and I-FABP.

Several previous studies have investigated the association between HIV infection, loss of gut barrier integrity, and immune activation using plasma markers of innate immune activation (soluble CD14 [sCD14]) and of elevated gut epithelial turnover (intestinal fatty acid-binding protein [I-FABP]) (49–54). We measured these markers in our GI study participants and additional samples from an unrelated HIV uninfected healthy participants not undergoing GI investigation (Figure 8). Compared with these non-GI controls, we found elevated sCD14 (*P* < 0.0001) and I-FABP (*P* < 0.0001) levels in all GI participants, and we detected no significant differences between HIV-infected and -uninfected individuals in the GI cohort (Figure 8A). To confirm this, we measured additional plasma markers of inflammation (IL-6, TNF-α), macrophage activation (sCD163), a further marker of intestinal barrier break down Zonulin, and the antiinflammatory soluble IL-33 receptor sST2 (Supplemental Figure 5); we found no significant differences between HIV infected and uninfected individuals from the GI cohort.

Taking all individuals together, we observed a positive correlation between sCD14 and I-FABP, consistent with a mechanistic link between elevated gut epithelial turnover and systemic innate immune activation in this cohort of subjects with GI complications (*r* = 0.4; *P* = 0.002) (Figure 8B). However, there was no correlation between gut CD4^+^ T cell frequency and matched plasma I-FABP or sCD14 levels, and there was no correlation with activation of blood CD4^+^ T cells (Figure 8C). When individuals were grouped by HIV status, we also found no correlation between these plasma markers and GI CD4^+^ T cell frequency or blood CD4^+^ T cell activation in individuals with suppressed viremia (Table 2). There was a significant negative association between I-FABP and GI CD4^+^ T cell frequency, and there was a nonsignificant positive association with blood T cell activation in the small number of viremic subjects tested.

Taken together, these data suggest that gut barrier breakdown and systemic innate immune activation are likely to be general markers of GI distress, are not unique to HIV-infected individuals, and are unlikely to occur as a direct consequence of GI CD4^+^ T cell depletion. Importantly, they also imply that systemic T cell activation does not occur as a direct result of GI barrier breakdown, at least in virally suppressed individuals.

## Discussion

This study presents clinical and immunological changes associated with HIV infection in a large cohort of individuals attending a tertiary GI clinic for investigation. We found an overall HIV prevalence of 32%, that reached > 60% in younger women, with 83% of all PLWH being on fully suppressive ART. Flow cytometric analysis of gut biopsies from more than 200 GI participants revealed severe depletion of CD4^+^ T cells associated with HIV — in particular, within the small intestine, compared with colon and blood compartments. This was most severe in the small intestine and was not reversed by full plasma viral suppression and long-term ART treatment. Greater CD4^+^ T cell depletion within the small intestine compared with the colon of PLHW was confirmed in situ, by fluorescence microscopy. Interestingly, higher CD4^+^ T cell levels within the large intestine in participants under full plasma viral suppression was accompanied by more abundant HIV-infected CD4^+^ T cell and CD68 macrophages, as shown by HIV-p24 protein detection. This implies that target cell availability may play an important role in governing the HIV viral reservoir in the GI tract. Importantly, although the level of both EFV and FTC appeared to lower in the gut than the plasma, we found no evidence of discordant ARV drug penetrations between the duodenum and colon, suggesting that ARV levels do not explain these differential effects. CD4^+^ T cell frequency in the gut of PLWH was inversely correlated with activation of circulating T cells, a biomarker that correlates strongly with HIV disease progression (55). This was not observed in HIV-uninfected individuals, despite elevated levels of plasma markers of innate immune activation and increased gut barrier permeability, such as sCD14 or I-FABP, respectively. Previous studies examining HIV-associated gut pathology have observed elevated levels of these and other plasma markers in relation to HIV^–^ healthy controls (49, 52, 56, 57). Uniquely, as these data are generated from individuals all attending a clinic for intestinal complications; this demonstrates that such markers are elevated in individuals with GI complications in general and not just those with HIV infection. It has been argued that the observation of increased gut permeability with immune activation in PLWH suggests a mechanistic link between HIV infection, increased gut permeability, and the persistent immune activation that drives HIV progression (54, 57–59). Here we show, however, that systemic immune activation of both CD4 and CD8 T cells, at least, is not linked to gut permeability per se, but rather to depletion of GI CD4^+^ T cells. However, we cannot rule out that sustained GI tract damage and microbial translocation may also contribute to persistent systemic immune activation. To our knowledge, this is the largest study of GI participants recruited within a high HIV prevalence area in Sub-Saharan Africa and unequivocally identifies the lack of immune reconstitution throughout the intestinal mucosa as a persistent problem in PLWH despite long-term ART.

Although this study was not powered to determine differences in clinical presentation in PLWH, we observed a significant increase in biliary pathologies in this group. Although more work would be needed to explore this further, it is in line with previous studies (60) and suggests that HIV infection precipitates cholangiopathy (61, 62) despite plasma viral suppression in most individuals. In addition, we detected a low frequency (<3%) of inflammatory disorders, including bowel diseases, compared with non-African cohorts undergoing clinically indicated GI investigation (39). This underlines the potential differences between these and Sub-Saharan African GI cohorts and demonstrates the importance to study HIV gut pathology in relevant populations for better representation of the HIV epidemic.

Although ART has successfully decreased HIV-associated morbidity and mortality (63, 64), we found that virally suppressed HIV infected participants in our cohort presented a median of 12 years earlier to the GI clinic than HIV-uninfected attendees, predominantly with noninfectious comorbidities. This early GI pathology associated with HIV could imply premature immune aging in this group, irrespective of plasma viral suppression (15), that precipitates GI complications. However, we cannot rule out that HIV-infected participants are better linked to care than uninfected participants, which can result in earlier clinical investigation.

The severe CD4^+^ T cell depletion observed throughout the large and small intestine is consistent with early observations in White cohorts (3, 4) and further underpins the lack of immune reconstitution in the gut (65). We observed a more pronounced CD4^+^ T cell depletion in the duodenum compared with the colon. This could be due to an increased abundance of lymph node structures, such as peyer’s patches, cryptopatches, or isolated lymphoid follicles (ILF) in the lamina propria of the large intestine and requires further validation, ideally in colon and duodenum samples from the same participants. Strikingly, we observed absolutely no correlation between the duration of ART and GI CD4^+^ T cells, suggesting that these cells are either continually being depleted or that the CD4 niche in this tissue has been irreparably damaged and cannot be repopulated. Recent data show that intestinal lamina propria CD4^+^ T cells are essential for regulating intestinal stem cells and epithelial cell differentiation in the crypt base (66, 67). The blood compartment showed a trend toward CD4 reconstitution, which may indicate late ART initiation in the majority of HIV-infected GI participants (68) and further emphasize that intestinal CD4 levels, in particular within the small intestine, do not recover despite long-term ART (65) in contrast to treatment initiation during early acute HIV infection (69).

The observation of more pronounced CD4^+^ T cell depletion in the duodenum compared with the colon was shown by both flow cytometry and histology. This is a potentially important observation, since, for practical reasons, many researchers studying the impact of HIV on the GI tract have limited their study to the colon. As CD4 loss was less pronounced in this compartment and appears to be somewhat better restored by ART, limiting observations to the colon may miss key aspects of persistent GI pathology in PLWH. It is important to note, however, that we were unable to obtain paired duodenum and colon samples from the sample individuals. It is therefore possible that compartment-specific differences may in part be explained by differences in the HIV patients presenting to the clinic for either upper or lower GI tract endoscopy.

Although the life expectancy of PLWH has been prolonged by the improvement in ART over the last few years (70), the gut has been implicated as a potential reservoir for ongoing HIV-1 replication in the setting of ART suppressed viremia (26). We tested if discordant ARV levels between duodenum and colon mucosal tissue, with plasma matched controls, could explain the difference in CD4^+^ T cell depletion. We found no evidence of compartment-specific ARV levels, but we found that duodenal CD4^+^ T cell depletion occurs in confirmed ARV-detected mucosal tissue, though this was based on a few individuals available. Thus, reduced ARV levels in mucosal tissue compared with plasma levels (14) does not necessarily explain the lack of CD4^+^ T cell reconstitution in this cohort. One caveat remains: comparing ARV levels in plasma to homogenized gut tissue cells may be suboptimal instead of using tissue matched PBMCs and normalized by cell numbers. However, previous studies have showed a correlation between plasma and PBMC ARV levels (71–76). Whether ongoing viral replication could be linked to reduction or absence of ARVs in gut mucosal tissue (33) remains to be tested in this cohort. Finally, HIV-p24 detection in both the small and large intestine, despite full viral suppression, may also be linked to ARV drug pharmacokinetics (77, 78). In plasma samples, we found evidence of treatment failure in which 3 ARVs were detected in 40% of viremic patients; this highlights the vulnerability of this population, where ART adherence over a long period of time is challenging (79) and also could accelerate GI complications in this region (80).

Our GI cohort underpins the central role of the intestine in HIV-1 pathology and demonstrates poor restoration of gut mucosal immunity, in particular within the small intestine, by otherwise effective ART. This ongoing GI study cohort presents a unique opportunity to further reveal the unknown mechanisms of GI impairment that are central to both HIV pathogenesis and its associated comorbidities in the growing population of individuals receiving ART in Sub-Saharan Africa, a distinct and critical population that is under studied. Future elected mucosal tissue sample collection from matched large and small intestinal tissue sites, combined with better characterized participants with available long-term clinical information from this region, will be a key resource for more in depth understanding of the mechanisms underlying HIV pathology in the GI tract.

## Methods

### Study participants and sample collection.

Patients presenting to the GI surgical unit of IALCH were recruited into this study after obtaining written informed consent. IALCH is an 846-bed central and tertiary hospital in the eThekwini district of KZN Province in South Africa. It provides regional and tertiary services to KZN, as well as referrals from the neighboring Eastern Cape Province. Duodenum, colon, and rectum pinch biopsies with participant-matched blood samples were obtained during endoscopy, endoscopic retrograde cholangiopancreatography (ERCP), and colonoscopy procedures. Blood samples were also obtained from a healthy HIV-uninfected volunteer study cohort (42). Clinical information, including HIV status and demographic details of these participants, was collected using a structured questionnaire. HIV status was confirmed using the COBAS TaqMan HIV-1 Test (Roche). Full blood count and viral load were done using the Sysmex XE-5000 Automated Hematology Analyzer and NUCLISENS EASYQ HIV-1 (BioMerieux), respectively.

### Mononuclear cells isolated from blood and gut.

Blood was collected in BD vacutainers with sodium heparin (Becton Dickinson). PBMCs were isolated using the Ficoll-Histopaque 1077 (Sigma-Aldrich) density gradient centrifugation at 2000 revolution per minute (RPM) for 20 minutes, at room temperature (25 ºC). Gut biopsies (2–4 pinches) were removed by the operating GI surgeon and transported to the laboratory in cold PBS (pH 7.2). The PBS was decanted from the tubes containing the gut biopsies, which are about 5–8 mm, and they were incubated in epithelial strip buffer (PBS [Sigma Aldrich, lot number P4417-100TAB]; 0.5M EDTA [ThermoFisher Scientific, catalog AM9261]; 1M DTT [Sigma Aldrich, catalog 43816]; FBS [Cytiva, catalog SV30160.03IR] and penicillin/streptomycin [Lonza, catalog DE17-602E]) in a 37°C water bath for 10 minutes, with occasional agitation. Thereafter, the epithelial strip buffer was removed, and the tissues were digested in a buffer containing Collagenase-D (0.5 mg/mL; Roche) and DNase-I (20 μg/mL; Sigma-Aldrich) for 30 minutes in a 37°C water bath with occasional agitation. Digested tissue was passed through a 70 μM cell strainer to isolate the cells, and these cells were washed with PBS.

### Flow cytometry.

PBMC and gut mononuclear cells were stained with antibody mixtures for a minimum of 20 minutes at room temperature. Cells were washed twice with PBS and acquired immediately or fixed in 2% paraformaldehyde for later acquisition within 24 hours. The BD FACSAria Fusion flow cytometer was used for acquisition of sample data. A minimum of 100,000 total events was collected for each sample, and data analysis was done using FlowJo software (version 9.7.2 or higher, TreeStar Inc.). The antibody mixture consists of Live/Dead Fixable Near-IR Cell Marker (Invitrogen) and an optimized antibody cocktail for the different phenotypic staining that included the following antibodies anti-CD45 (BD Biosciences, 560777) anti-CD3 (BioLegend, 317330) anti-CD4 (BD Biosciences, 564651), anti-CD8 (BD Biosciences, 563795), anti-HLA-DR (BioLegend, 307606).

### Measurement of drug levels in gut and plasma.

The concentration of ARV drugs was simultaneously quantified in plasma and gut tissue samples using LC-MS/MS. The tissue samples were homogenized using a TH Omni international homogenizer/soft tissue probe combination (Omni-International) with a short pulse (15 seconds) in 500 μL of cold acetonitrile/water (1:1, v/v) solution and then processed for analysis. The drug concentration data were normalized to the average mass of the tissue sample set and used a tissue density of 1.06 g/mL to relate tissue weight to tissue volume as previously described (75). The ARV drug analytes were extracted from calibration standards (STDs), quality control (QCs) samples, and study samples using protein precipitation followed by LC-MS/MS analysis. Human plasma was used as a surrogate biological matrix for the preparation of quantitative STDs and QCs, due to the limited availability of control (healthy donor/drug-free) gut tissue sample. Matrix effect studies to compare drug quantitation between spiked plasma and homogenized gut tissue samples were performed and showed equivalency with less than 15% variability in analyte peak areas.

The extracted analytes were chromatographically separated on an Agilent Zorbax Eclipse Plus C18 (2.1 × 50 mm, 3.5 μm) HPLC column, at 40°C, using gradient elution with a combination of mobile phase A, which consisted of water with 0.1% formic acid, and phase B, which consisted of acetonitrile with 0.1% formic acid. A sample volume of 20 μL was injected, the flow rate was set to 0.2 mL/minute, and the total run time was 20 minutes. All ARV drug analytes were analyzed in the positive ionization mode, using 6-Aminonicotinic acid (6-ANA) as an internal standard — except for EFV, which was analyzed in the negative ionization mode using deuterated EFV (EFV-d5) as an internal standard. Data were acquired using selected reaction monitoring (SRM) and processed using AB Sciex Analyst Software, version 1.6.2, on an AB Sciex Triple Quad 5500 mass spectrometer.

### Histology and fluorescent IHC.

Multiplex fluorescent IHC staining was performed using the Opal 4-Color Manual IHC Kit 50 Slides (PerkinElmer) as directed by the manufacturers. Duodenum and colon tissue samples fixed in 4% formalin between 2 days and a month were paraffin embedded. Sections (4 um) were cut on glass slides, and the slides were baked at 60°C overnight. Then, the combined process of deparaffinization, rehydration, and antigen retrieval of the tissue sections was done using the PT-Link Pre-Treatment instrument (Dako) and 1× Envision Target Retrieval Solution, High PH (Dako). Then, slides were incubated for 1 minute in distilled water and equilibrated in 1× EnVision FLEX Wash Buffer (Dako) for 5 minutes at room temperature. Next, the slides were incubated in Peroxidase blocking solution (PerkinElmer) for 10 minutes and washed in 1× wash buffer (Dako) immediately at room temperature. The slides were then incubated in Bloxall blocking solution (PerkinElmer) for 10 minutes, and next in primary antibody-1 for 30 minutes at room temperature. Slides were washed for 5 minutes in 1× wash buffer and incubated in Secondary Opal Polymer Horseradish Peroxidase (HRP) Mouse and Rabbit (PerkinElmer) for 30 minutes. The Opal Polymer HRP is recommended for human tissues with mouse or rabbit primary antibodies. Then, the slides were washed twice in 1× wash buffer and drained, and the sections were incubated in Opal Fluorophore (PerkinElmer) diluted 1:100 in amplification diluent (PerkinElmer) working solution for signal amplification at room temperature for 10 minutes. The slides were then washed for 5 minutes in 1× wash buffer at room temperature. Afterward, antibody stripping via microwave treatment was done by placing the slides in a slide jar with prewarmed buffer 1× AR6 (PerkinElmer). The jar was loosely covered and placed in a microwave for 2 minutes at 100% power, 10 minutes at 50% power, and 5 minutes at 20% power. Slides were allowed to cool in the dark by placing the slide jar on ice for 20 minutes, and the slides were rinsed in distilled water, followed by incubation in the 1× wash buffer for 5 minutes to equilibrate. The microwave step strips the primary-secondary-HRP complex and allows the introduction of the next primary antibody. For the detection of the next target (primary antibody-2; anti-CD4, clone 4B12, Dako), the protocol was restarted at the blocking step using Bloxall blocking solution (PerkinElmer) for 10 minutes. After the third target was detected (primary antibody-3; anti-CD68, clone KP1, Dako), a working solution of DAPI (PerkinElmer) was applied to the sections as the nuclear counterstain for 5 minutes. The slides were washed in 1× wash buffer for 5 minutes, then in distilled water for 5 minutes and drained. Then, the sections were coverslip with Fluorescence Mounting Medium (Agilent Technologies, S302380-2) and the edges of the coverslip were sealed with nail polish. Slides were stored in a humidity chamber at 2°C–8°C until images are acquired.

The unlabeled primary antibodies used in this study were anti-HIV p24 (clone Kal-1, Dako) used as the first antibody in the staining cycle, diluted 1:10 in antibody diluent (PerkinElmer), and anti-CD4 (clone 4B12, Dako), which was the second antibody in the staining cycle. It was pre-mixed, followed by anti-CD68 (clone KP1, Dako) as the third antibody in the cycle, diluted 1:200 in antibody diluent (PerkinElmer). The fluorophores were FITC (FP1487001KT, PerkinElmer) for anti-HIV p24 (clone Kal-1, Dako), Texas-Red (product no. FP1488001KT; PerkinElmer) for anti-CD4 (clone 4B12, Dako), and Cy5 (product number FP1497001KT; PerkinElmer) for anti-CD68 (clone KP1, Dako) signal generation. These fluorophores were diluted 1:100 in amplification diluent. Images of the tissue sections were acquired using the TissueFAXS software (TissueGnostics) connected with a Zeiss Axio Observer Z1 inverted microscope (Olympus). The quantitative analysis of the cells of the different phenotypes within the images was done using the TissueQuest quantitation software (TissueGnostics). Isotype and nonantibody controls were included in the experiment to rule out nonspecific staining and autofluorescence.

### Measurement of blood and gut HIV-1 DNA using ddPCR.

Total PBMC and duodenum and colon tissue mononuclear cells from study participant were subjected to DNA extraction using DNeasy Blood & Tissue Kits (QIAGEN). Total HIV-1 DNA and host cell concentrations in the DNA extracts were estimated using Bio-Rad ddPCR, using primers and probes covering HIV-1 5′ LTR-gag HXB2 coordinates 684–810 (81) (forward primer 5′-TCTCGACGCAGGACTCG-3′, reverse primer 5′-TACTGACGCTCTCGCACC-3′ probe/56-FAM/CTCTCTCCT/ZEN/TCTAGCCTC/31ABkFQ/, and human RPP30 gene (82) forward primer 5′-GATTTGGACCTGCGAGCG-3′, reverse primer 5′-GCGGCTGTCTCCACAAGT-3′, probe/56-FAM/CTGACCTGA/ZEN/AGGCTCT/31ABkFQ/). ddPCR was performed using the following thermocycler program: 95°C for 10 minutes, 45 cycles of 94°C for 30 seconds, and 60°C for 1 minutes, 72°C for 1 minute. The droplets were subsequently read by the Bio-Rad QX100 droplet reader, and data were analyzed using QuantaSoft software (Bio-Rad).

### Markers of microbial translocation and inflammation.

The concentration of some of the plasma markers was measured by ELISA using Human sCD14 DuoSet ELISA (R&D Systems, DY383), Human FABP2/I-FABP DuoSet ELISA (R&D Systems, DY3078), and Human Zonulin/Haptoglobin ELISA kit (ARP American Research Products, E01Z0004) per manufacturer’s instructions. Also, Milliplex multiplex assays using Luminex were used to determine the concentration of soluble ST2 and CD163 using the Human Cardiovascular Disease Magnetic bead panel 5 (Milliplex, HCVD5MAG-67K), per manufacturer’s instructions. The concentration of IL-6 and TNF-α was determined using the Human TH17 magnetic Bead Panel (Milliplex, HTH17MAG-14K) per manufacturer’s instructions.

### Statistics.

Descriptive and categorical data were represented as medians with interquartile ranges and minimum and maximum values, and differences were analyzed using the Mann-Whitney *U* test. The Kruskal-Wallis test was performed for multiple comparisons between HIV-uninfected and each of the HIV-1–infected groups. Bivariate correlations were determined by the Spearman’s rank correlation test. A multivariate logistic regression model was used to estimate the association between HIV-1 and GI comorbidities at a 95% CI. Correlations were calculated using Pearson correlations assuming Gaussian distributions. Statistical analyses were performed using GraphPad Prism (version 8) and STATA (version 15) software, and *P* <0.05 was considered statistically significant.

### Study approval.

This study was approved by the Biomedical Research Ethics Committee (BREC) of the University of KwaZulu-Natal (REF: BE 021/13) following its ethical standards and with the 1964 Declaration of Helsinki and its later amendments. Patients presenting to the GI surgical unit of IALCH were recruited into this study after obtaining written informed consent.

## Author contributions

OEA and AS performed experiments. FN and NM recruited study participants. FN and NM consented participants in the GI ward. DR and FK coordinated human specimens. OEA, AS, AN, RF, YZ, KG, NH, and KR contributed to experimental work. FGM, VTM, and FA contributed surgical human tissues samples. NM, FN, FK, and TN contributed sample collection. HNK, OEA, and AL prepared the manuscript. TN, JZP, JHA, and AL provided intellectual input. HNK conceptualized and provided funding.

## Supplementary Material

Supplemental data

## Figures and Tables

**Table 1:**
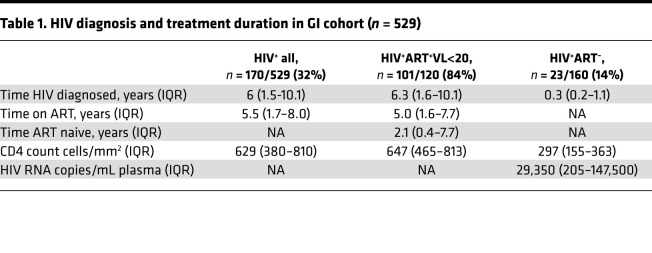
HIV diagnosis and treatment duration in GI cohort (*n* = 529)

**Table 2:**
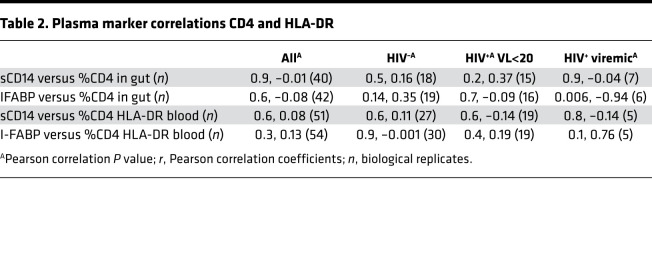
Plasma marker correlations CD4 and HLA-DR

## Version 1 10/07/2021

In-Press Preview

## Version 2 11/22/2021

Electronic publication
