# Supplementary material for: Irreversible depletion of intestinal CD4+ T cells is associated with T cell activation during chronic HIV infection
Source: JCI Insight. 2021 Nov 22;6(22):e146162. doi: 10.1172/jci.insight.146162 (PMC8663780; doi:10.1172/jci.insight.146162)
Supplement: Supplemental data [file jciinsight-6-146162-s143.pdf]

Supplementary Figure and Tables for Asowata O et al

'Irreversible depletion of intestinal $CD4^+$ T-cells is associated with T-cell activation during chronic HIV infection'

Supplemental Table 1 Demographics and TB treatment status (%) in GI cohort (n=529).

Supplementary Figures 1-5.

Supplemental Table 1. Demographics and TB treatment status (%) in GI cohort (n=529).

	All, n=529/529 (100%)	HIV$^-$, n=359/529 (68%)	HIV$^+$ all, n=170/529 (32%)	*HIV$^+$ART$^+$, n=102/120 (85%)	HIV$^+$ART$^-$, n=24/160 (15%)
**Gender**					
Female	62	57	74	77	65
Male	38	43	26	23	35
**Ethnicity**					
Black	64	53	86	85	78
Indian	24	30	12	14	17
Coloured	5	7	1	0	4
Caucasian	7	9	1	1	0
Other	0	0	0	0	0
**Procedure**					
Colonoscopy	37	42	31	31	22
Endoscopy	36	38	30	34	22
ERCP	28	22	39	36	57
**TB infection**					
Active TB	2	2	2	3	0
**Previous TB**					
Previous TB	11	4	24	27	9
**TB Treatment**					
^Current on treatment	10	8	13	14	0
**Starting ART at diagnosis**					
Starting ART at diagnosis	NA	NA	NA	38	NA

^Rifampin/Isoniazid/Pyrazinamide/Ethambutol. *Undetectable viral load (VL<20).

Suppl Figure 1 (Related to Figure 1).

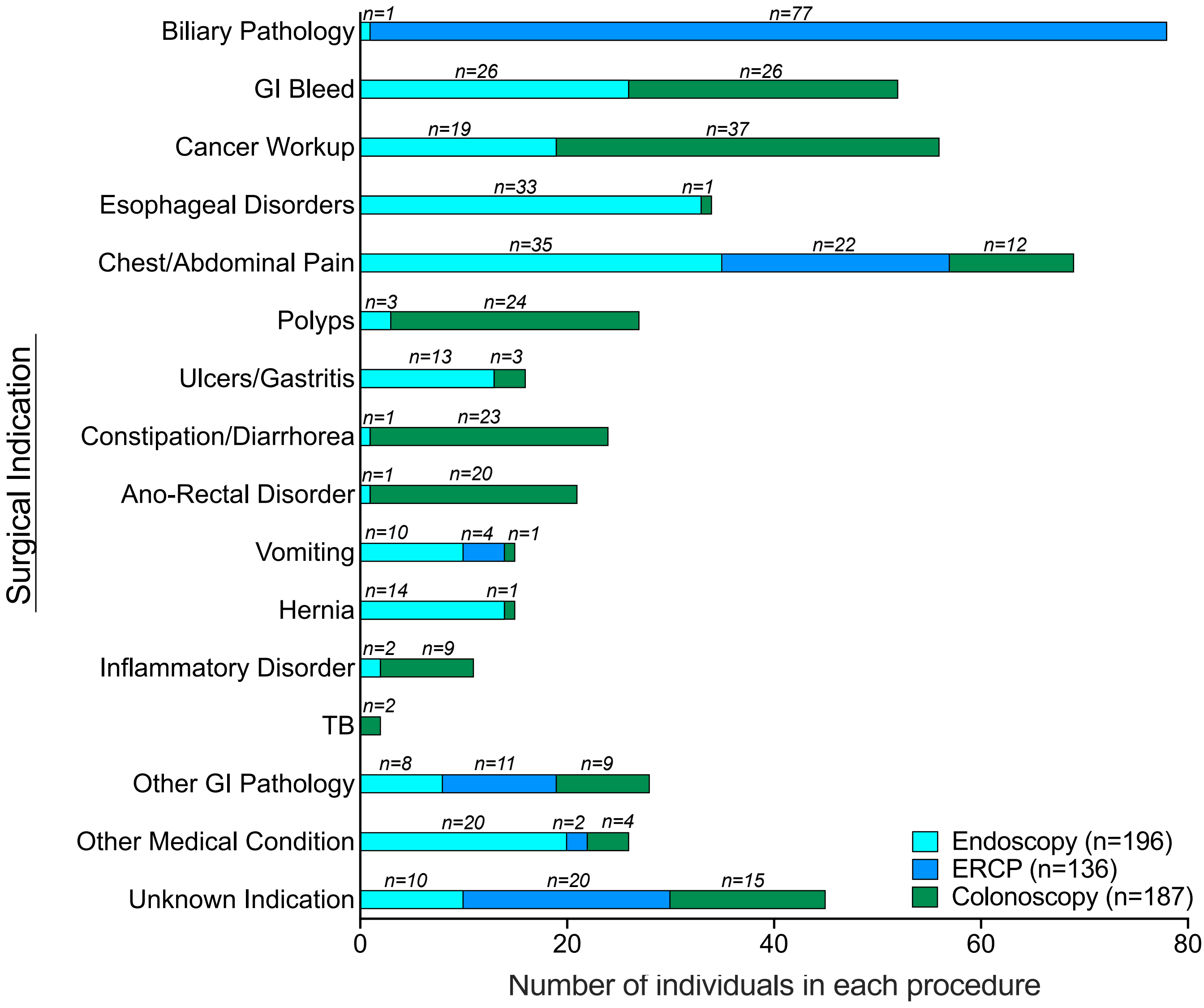

Supplementary Figure 1 (related to Figure 1). Surgical indication of participants presenting to the GI clinic stratified by procedures (Endoscopy, Colonoscopy, ERCP) (n=519). The number or patients for each indication/procedure combination is listed above each bar. 'Unknown' are patients who presented to the GI clinic with no surgical indication.

Suppl Figure 2 (Related to Figure 2).

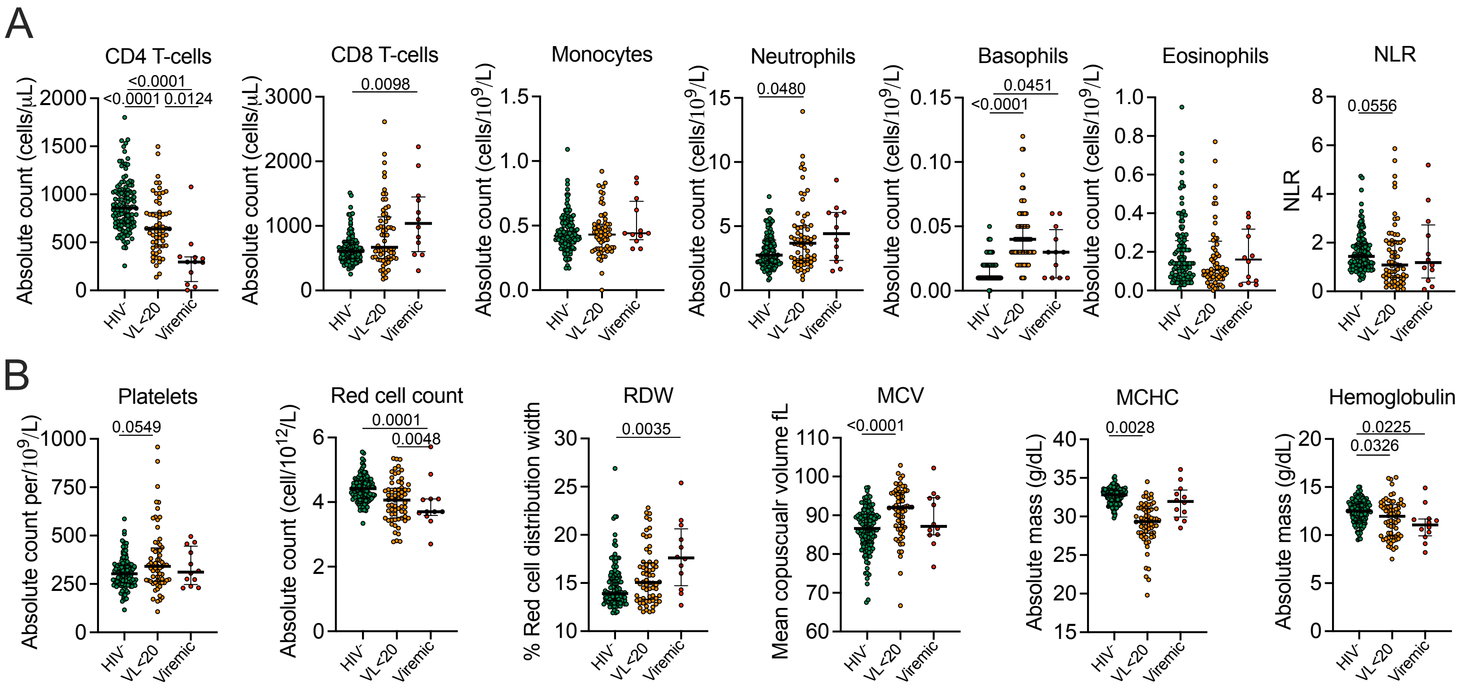

Supplementary Figure 2 (related to Figure 2). Full blood counts of study participants stratified by HIV status and viral load. P-values by Mann-Whitney U test.

Suppl Figure 3 (related to Figure 5)

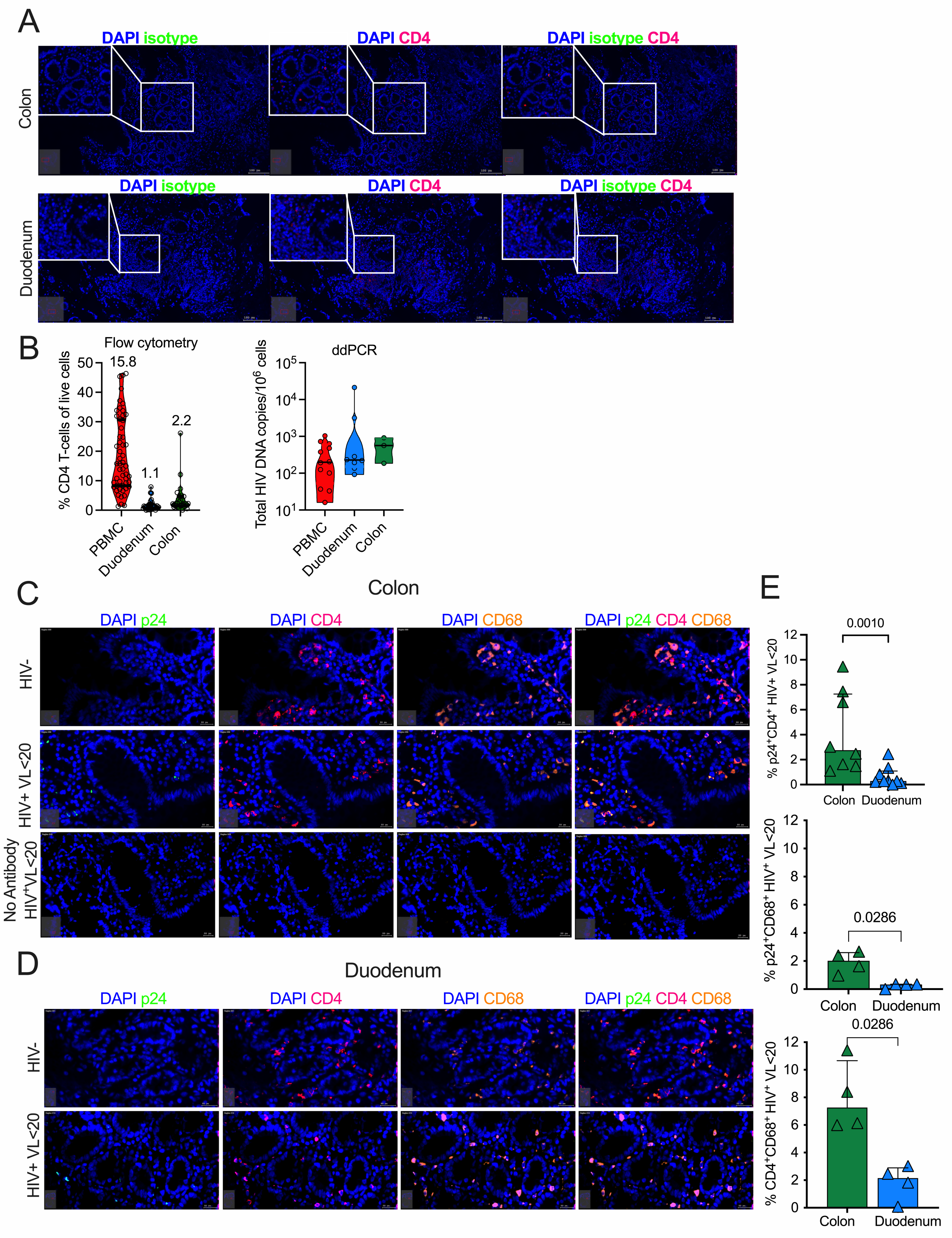

Supplementary Figure 3 (related to Figure 5). HIV-1-p24 detection in the gut mucosa in ART treated individuals. A. Representative fluorescent immunohistochemistry image of colon and duodenum tissue sections from study participant for HIV-1-p24 isotype controls (green) and CD4 T-cells (red) proportion of total cells stained with DAPI (blue) from HIV infected participants. Bars, 100 μm and 20 μm for large and inserted images respectively. **B**. % CD4 T-cells of live cells from PBMC, duodenum and colon of selected study participants; comparison of HIV-1 DNA in the blood to the duodenum and colon using digital droplet PCR (ddPCR). **C.** Representative fluorescent immunohistochemistry image of colon tissue sections from study participant for HIV-p24 (green), CD4 T-cells (red) and CD68 (orange) proportion of total cells stained with DAPI (blue) from HIV uninfected and HIV infected plasma virally suppressed participants, with no antibody controls included. Bars, 20 μm for all images. **D.** Representative fluorescent immunohistochemistry image of duodenum tissue sections from study participant for HIV-p24 (green), CD4 T-cells (red) and CD68 (orange) proportion of total cells stained with DAPI (blue) from HIV uninfected and HIV infected plasma virally suppressed participants. Bars, 20 μm for all images. **E**. Quantification of HIV-p24, CD4 T-cells and CD68 cells proportion of total cells in colon (green) and duodenum (blue) stained with DAPI from HIV infected virally suppressed participants. P-values by Mann-Whitney U test.

Suppl Figure 4 (Related to Figure 6).

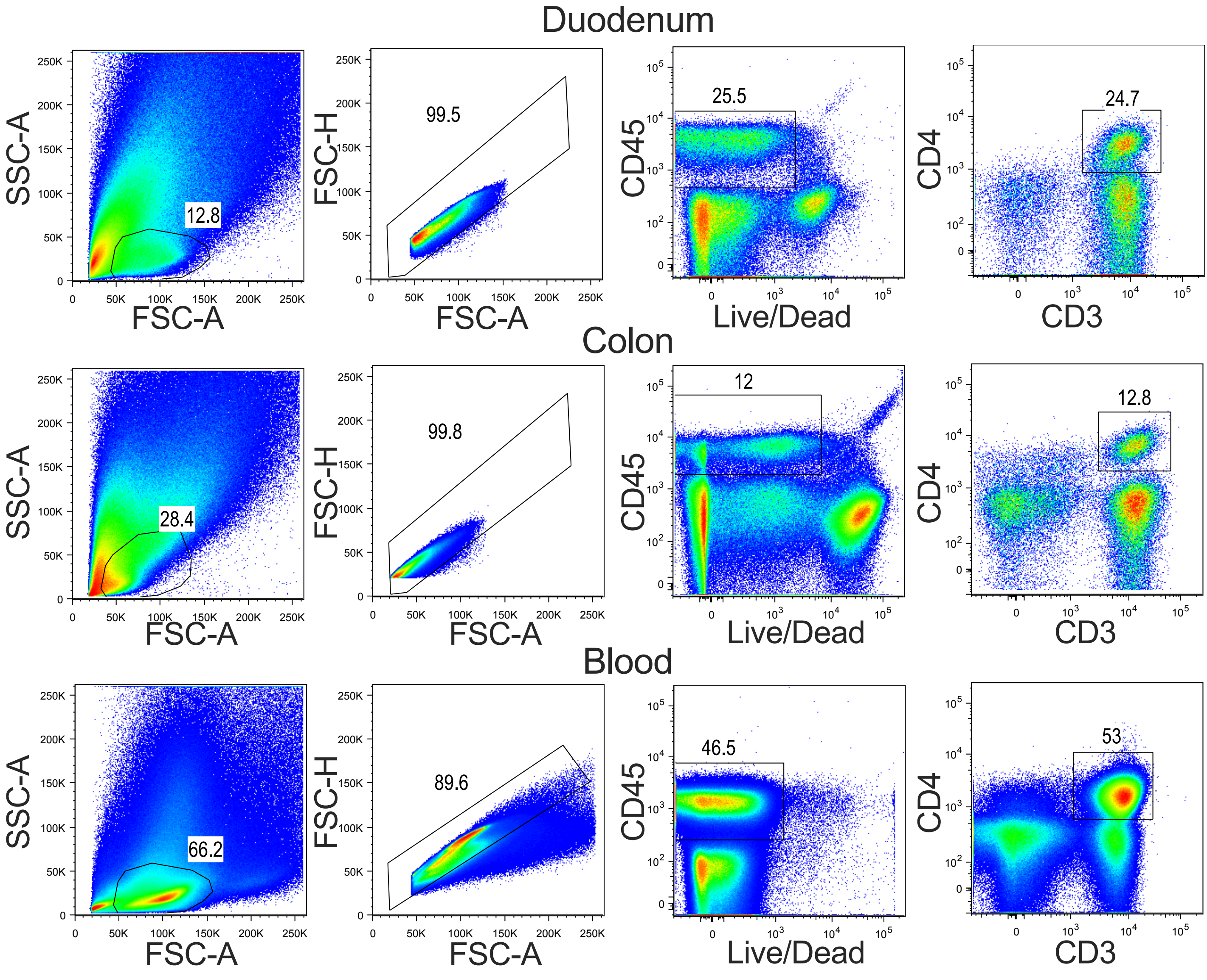

Supplementary Figure 4 (related to Figure 7). Flow cytometry gating strategy for duodenum, colon and blood T-cells.

Suppl Figure 5 (related to Figure 8)

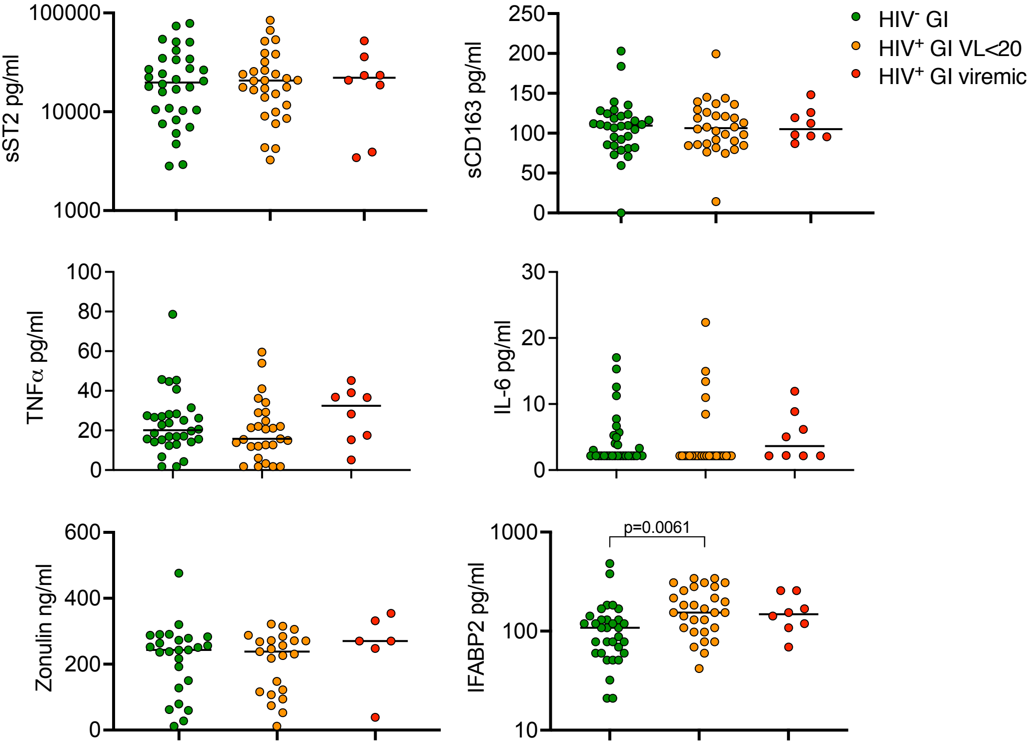

Supplementary Figure 5 (related to Figure 8). Innate markers of inflammation and immune activation stratified by HIV and viral load status. P-values by Mann-Whitney U test.
